# Virus perception at the cell surface: revisiting the roles of receptor‐like kinases as viral pattern recognition receptors

**DOI:** 10.1111/mpp.12816

**Published:** 2019-05-16

**Authors:** Ruan M. Teixeira, Marco Aurélio Ferreira, Gabriel A. S. Raimundo, Virgílio A. P. Loriato, Pedro A. B. Reis, Elizabeth P. B. Fontes

**Affiliations:** ^1^ National Institute of Science and Technology in Plant–Pest Interactions, Bioagro, Universidade Federal de Viçosa Viçosa Minas Gerais 36570‐000 Brazil; ^2^ Departament of Biochemistry and Molecular Biology Universidade Federal de Viçosa Viçosa Minas Gerais 36570‐000 Brazil; ^3^ Agronomy Institute, Universidade Federal de Viçosa Campus Florestal Florestal Minas Gerais 35690‐000 Brazil

**Keywords:** begomovirus, NIK1‐mediated antiviral signalling, NSP‐interacting kinase, PAMP‐triggered immunity, pattern recognition receptor, PRR, receptor‐like kinase, viral PAMPs

## Abstract

Activation of antiviral innate immune responses depends on the recognition of viral components or viral effectors by host receptors. This virus recognition system can activate two layers of host defence, pathogen‐associated molecular pattern (PAMP)‐triggered immunity (PTI) and effector‐triggered immunity (ETI). While ETI has long been recognized as an efficient plant defence against viruses, the concept of antiviral PTI has only recently been integrated into virus–host interaction models, such as the RNA silencing‐based defences that are triggered by viral dsRNA PAMPs produced during infection. Emerging evidence in the literature has included the classical PTI in the antiviral innate immune arsenal of plant cells. Therefore, our understanding of PAMPs has expanded to include not only classical PAMPS, such as bacterial flagellin or fungal chitin, but also virus‐derived nucleic acids that may also activate PAMP recognition receptors like the well‐documented phenomenon observed for mammalian viruses. In this review, we discuss the notion that plant viruses can activate classical PTI, leading to both unique antiviral responses and conserved antipathogen responses. We also present evidence that virus‐derived nucleic acid PAMPs may elicit the NUCLEAR SHUTTLE PROTEIN‐INTERACTING KINASE 1 (NIK1)‐mediated antiviral signalling pathway that transduces an antiviral signal to suppress global host translation.

## Introduction

All plant cells are naturally and frequently exposed to microorganisms. To cope with invading pathogens, plant cells evolved a sophisticated immune system under constant pressure for dominance over the pathogens’ virulence strategies, which in turn coevolved to escape the host recognition system (Jones and Dangl, 2006). The first layer of the plant immune system is represented by the pattern recognition receptors (PRRs) at the cell surface, which recognize either conserved signature molecules produced by the pathogens, designated pathogen‐associated molecular patterns (PAMPs), or endogenous damage/danger signals associated with pathogen invasion, designated danger‐ or damage‐associated molecular patterns (DAMPs) (Choi and Klessig, 2016; Ma *et al.*, 2016). The sensing of PAMPs by PRRs activates PAMP‐triggered immunity (PTI), leading to a rapid, non‐specific response to a broad range of pathogens (Ma *et al.*, 2016). To counterattack this first layer of defence, adapted pathogens deliver virulence effectors in the host cell cytoplasm, which prevent the activation of PTI and elicit effector‐triggered susceptibility (ETS; Wang and Wang, 2018). In response, plant cells have evolved intracellular nucleotide‐binding leucine‐rich repeat (NLR) receptors, which recognize the virulence effectors in a highly specific manner to activate the second level of plant defence and are designated as effector‐triggered immunity (ETI; Jones and Dangl, 2006).

In plant–virus interactions, a common theme that has emerged from recent studies is that plants may also employ classical PTI to fight virus infection similarly to non‐viral pathogens (Iriti and Varoni, 2015; Korner *et al.*, 2013; Nicaise and Candresse, 2017; Niehl *et al.*, 2016). Activation of antiviral PRR pathways by viral PAMPs (VAMPs) has been extensively studied in mammals, and the mechanisms by which viral effectors manipulate PTI defences have been well characterized (Jensen and Thomsen, 2012; Yokota *et al.*, 2010). The mammalian Toll‐like receptors (TLRs) are classic examples of antiviral PPRs with similar receptor configuration to plant leucine‐rich repeats (LRR) receptor‐like kinases (RLKs), which function as PRRs and coreceptors in plant immunity (Botos *et al.*, 2011). Although these LRR‐RLK receptors have collectively been shown to sense a variety of PAMPs, only viral infection‐sensing LRR‐RLKs are in the scope of this minireview.

### PTI‐based antiviral responses by receptor‐like kinases

**Figure 1:**
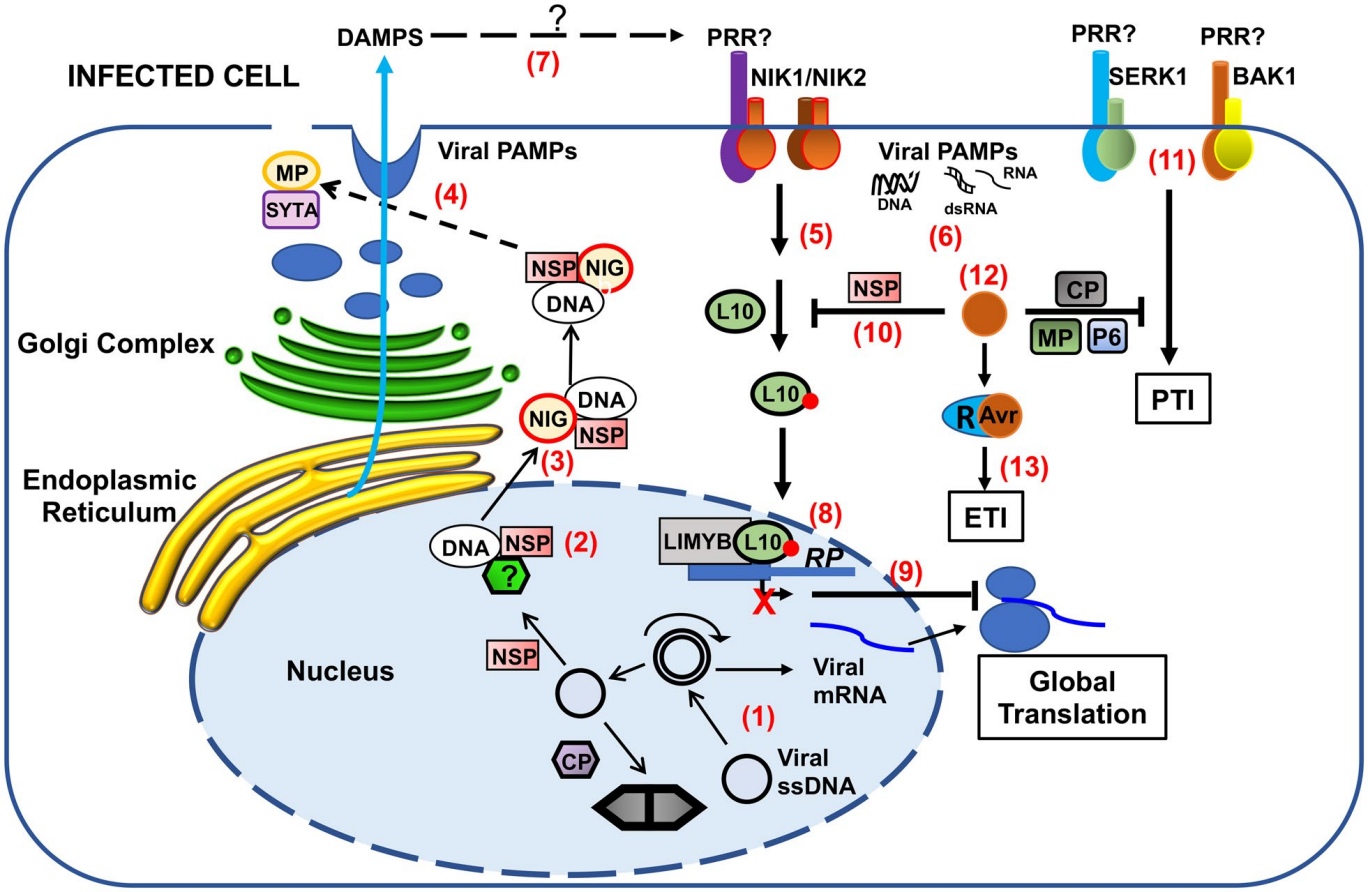
Activation of the NIK1‐mediated antiviral signalling and antiviral PTI. The viral single‐stranded DNA from begomoviruses replicates via double‐stranded DNA intermediates that are transcribed in the nucleus of infected plant cells (1). NSP binds to the nascent viral DNA and facilitates its movement to the cytoplasm via nuclear pores (2). At the cytosolic side, the NSP‐interacting GTPase (NIG) helps the release of NSP‐viral DNA complex from the nuclear pores into the cytoplasm (3) and then moves the viral DNA complex towards the cell periphery. MP associates with the endosomal synaptotagmin A (SYTA) and may interact directly with the NSP–viral DNA complex to move the viral DNA to plasmodesmata via an endocytic recyclizing pathway for the MP‐assisted cell‐to‐cell spread (4). In incompatible interactions, plant cells may elicit the translational control branch of the NIK1‐mediated antiviral signalling as an innate defence against DNA viruses (5). The mechanism of NIK1 transmembrane receptor activation is unknown, but we showed here that both RNA and DNA from infected plants activate the NIK1‐mediated antiviral signalling (6). As plant viruses depend on the insect‐vector‐induced mechanical injury for entry into the plant cells, endogenous DAMPs induced during infection may also activate the antiviral innate immune system (7). Upon activation, NIK1/NIK2 mediates the phosphorylation of RPL10 and subsequent translocation to the nucleus, where it interacts with LIMYB to fully repress the expression of translational machinery‐related genes [ribosome protein (RP) genes for instance] (8). Prolonged down‐regulation of translation‐related genes leads to global translation suppression, which also impairs viral mRNA (vmRNA) translation (9). In begomovirus–host compatible interactions, NSP binds to NIK1 and suppresses its activity, creating a favourable environment for virus infection (10). In the case of RNA and DNA viruses, replication and expression of viral genomes lead to the accumulation of non‐self DNA or RNA motifs (virus‐derived PAMPs, dsRNA, RNA and DNA), which may be recognized by PRRs that in turn heteromultimerize with co‐receptors (BAK1 or SERK1) to trigger antiviral PTI (11), which may also be activated by endogenous DAMPs (7). In any case, in RNA or DNA viruses, a successful infection implicates in the accumulation of viral effectors (e.g. CP from PPV and NSP from begomoviruses) to suppress PTI, leading to disease (12). In resistant genotypes, however, the resistance (R) proteins specifically recognize, directly or indirectly, the viral effectors, called avirulence (Avr) factors, activating ETI and conferring resistance (13). Adapted from Gouveia *et al.*, 2017.

For non‐viral pathogens, plant PTI and mammalian innate immune systems share similar activation mechanisms, including a similar structural configuration of PRRs and similar conserved PAMPs. However, plant antiviral PTI has been conceptually regarded as RNA silencing, in which viral dsRNAs are considered PAMPs, which activate Dicers as PRRs that can be suppressed by viral effectors (Calil and Fontes, 2017; Moon and Park, 2016). More recently, accumulated evidence has invoked the classic PTI initiated by transmembrane PRRs as part of the plant defence arsenal against viruses. Evidence for the classic PTI against plant viruses is based on experimental data of the betacarmovirus *Turnip crinkle virus* (TCV; Korner *et al.*, 2013; Yang *et al.*, 2010), the potyviruses *Soybean mosaic virus* (SMV; Liu *et al.*, 2011) and *Plum pox virus* (PPV; Nicaise and Candresse, 2017), the tobamoviruses *Tobaco mosaic virus* (TMV) and *Oilseed rape mosaic virus* (ORMV; Korner *et al.*, 2013), the alfamovirus *Alfalfa mosaic virus* (AMV) and the potexvirus *Potato virus X* (PVX; Iriti and Varoni, 2015), the caulimovirus *Cauliflower mosaic virus* (CaMV; Zvereva *et al.*, 2016), and the cucumovirus C*ucumber mosaic virus* (CMV; Kong *et al.*, 2018). Collectively, these studies provided several lines of evidence that the classical plant PTI limits virus infection similar to non‐viral pathogens. First, preactivation of PTI with non‐viral PAMPs confers resistance to virus infection, indicating that PTI‐induced immune responses confer protection against viruses (Iriti and Varoni, 2015). Second, it has been conceptually accepted that pathogens have to suppress PTI in order to successfully colonize a host. Growing evidence has shown that viruses are not exceptions, and viral suppressors of PTI have been identified recently, including the PPV coat protein (CP; Nicaise and Candresse, 2017), CaMV P6 (Zvereva *et al.*, 2016) and the movement protein (MP) from CMV (Kong *et al.*, 2018). PPV CP also functions as an Avr factor recognized by the RTM resistance in *Arabidopsis* (Decroocq *et al.*, 2009). Therefore, PPV CP may link the suppression of PTI with activation of ETI, as predicted by the zigzag evolutionary model of plant innate immunity (Jones and Dangl, 2006; Fig. 1). Finally, reverse genetics and overexpression studies with characterized PTI components have demonstrated that plants efficiently use PTI to limit viral infection (Kørner *et al*., 2013; Liu *et al.*, 2011; Nicaise and Candresse, 2017, Niehl, *et al.*, 2016; Yang *et al.*, 2010). An obvious caveat from these previous results is that no virus‐derived PAMP detected by a plant PRR has been identified, and hence antiviral PRRs remain to be described. Recently, both virus‐derived and synthesized double‐stranded (ds) RNAs have been shown to activate plant PTI and to restrict virus infection in an RNA‐silencing independent manner (Niehl, *et al.*, 2016; Fig. 1). This dsRNA‐induced PTI in plants requires the function of the LRR‐RLK coreceptor SOMATIC EMBRYOGENESIS RECEPTOR KINASE 1 (SERK1) linking virus perception to the membrane‐anchored immune receptor signalling.

The direct inactivation of plant PTI components has provided compelling evidence that transmembrane PRR‐mediated PTI operates against viruses in plants. Frequently, the membrane‐anchored immune RLKs and receptor‐like proteins (RLPs) depend on ligand (PAMP/DAMP)‐induced dimerization or oligomerization with coreceptors for activation (Ma *et al.*, 2016). The BRASSINOSTEROID INSENSITIVE1 (BRI1)‐ASSOCIATED KINASE 1 (BAK1) functions as a coreceptor that interacts with multiple PAMP‐interacting immune receptors (RLKs and RLPs) to assemble active PTI signalling complexes (Ma *et al.*, 2016). Like BAK1, other members of the subfamily II of LRR‐RLKs, including SERK1, SERK4/BKK1 (BAK1‐LIKE KINASE 1) and the NUCLEAR SHUTTLE PROTEIN‐INTERACTING KINASE 1 (NIK1), function in antiviral immunity (Gouveia *et al.*, 2017). Accordingly, inactivation of the PTI regulator BAK1 or BKK1 enhances susceptibility to RNA virus infection, demonstrating that they are required to build an effective defence against RNA viruses in *Arabidopsis *(Kørner *et al*., 2013; Yang *et al.*, 2010; Fig. 1). This assembled defence is more likely due to BAK1‐dependent PTI because crude viral extracts from infected plants induced several PTI marker responses in a BAK1‐dependent manner (Kørner *et al*., 2013). Likewise, *serk1* knockout lines are more susceptible to virus infection (Niehl, *et al.*, 2016), and the *Arabidopsis* double mutant *bak1‐5/bkk1* displays increased viral accumulation when inoculated with PPV (Nicaise and Candresse, 2017). Collectively, these results further substantiate the notion that plant PTI functions to combat virus infection similarly to non‐viral pathogens. These recent data have also implicated plasma membrane‐localized coreceptors of PRRs, such as BAK1, BKK1, and SERK1, in antiviral PTI, leaving an open question on how viruses, which are intracellular obligate parasites and are expected to deliver PAMPs intracellularly, are perceived extracellularly (Fig. 1).

### NIK1‐mediated antiviral immunity: from a PTI‐like activation to a somewhat distinct response

NIK1 was first identified as a virulence target of the nuclear shuttle protein (NSP) from the bipartite begomoviruses, *Geminiviridae* family (Mariano *et al.*, 2004; Silva *et al.*, 2017). NSP mediates the nuclear export of begomoviral DNA to the cytoplasm, a process facilitated by the NSP‐interacting GTPase (Carvalho *et al.*, 2008a, 2008b). NIK1 belongs to the LRR‐RLKs subfamily II, which includes the SERK clade, the NIK clade and a third clade of uncharacterized members (Zhang *et al.*, 2006). In phylogenetic analysis, NIK1 and NIK2 cluster together and separately from NIK3 (Sakamoto *et al.*, 2012). Although NIK1 is structurally related to SERKs and is also implicated in plant immunity, the mechanism by which NIK1 propagates an antiviral signal, and the resulting immune responses are entirely different from the BAK1/SERK3‐dependent PTI response against pathogens (Machado *et al.*, 2015). The mechanism of NIK1‐mediated defence is underscored by repression of translational machinery genes and suppression of global translation, as a new paradigm for plant antiviral immunity (Machado *et al.*, 2017; Zorzatto *et al.*, 2015).

As Ser/Thr kinase receptors, NIK1 is activated by autophoshorylation (Santos *et al.*, 2010). Several lines of evidence indicate that phosphorylation at the essential Thr‐474 residue within the A‐loop constitutes a critical regulatory mechanism for NIK1 activation. NIK1 undergoes autophosphorylation *in vitro* at conserved threonine residues, positions Thr‐468, Thr‐469 and Thr‐474 within the activation loop, and mediates substrate phosphorylation *in vivo* (Santos *et al.*, 2009). While phosphorylation at Thr‐469 negatively regulates NIK1 kinase activity, phosphorylation at positions Thr‐468 and Thr‐474 enhances and promotes kinase activation, respectively. Within the conserved activation loop of the members of the LRR‐RLK subfamily II, Thr‐468 and Thr‐474 align to the same positions as the conserved BAK1 residues Thr‐449 and Thr‐455, and SERK1 residues Thr‐462 and Thr‐468, which are intramolecular targets for BAK1 and SERK1 kinase activation (Shah *et al.*, 2001; Wang *et al.*, 2005; Yun *et al.*, 2009). Furthermore, replacement of NIK1 Thr‐474 with alanine impairs autophosphorylation and substrate phosphorylation activity *in vitro* (Santos *et al.*, 2009). Consistently, the ectopic expression of loss‐of‐function Thr‐474 mutants, such as T474A or the double mutant G4743V/T474A, did not reverse the enhanced susceptibility phenotype of NIK1 knockout lines, demonstrating that Thr‐474 autophosphorylation is required to transduce a defence response against begomoviruses (Santos *et al.*, 2009). Finally, the replacement of Thr‐474 with the phosphomimetic amino acid aspartate promotes constitutive activation of the NIK1‐mediated antiviral signalling (Santos *et al.*, 2009). Ectopic expression of T474D in *Arabidopsis* and tomato transgenic lines promotes RPL10 phosphorylation, causes repression of ribosomal protein genes, suppresses global translation, decreases viral mRNA association with actively translating polysome fractions and confers resistance to begomoviruses, as readouts of NIK1‐mediated antiviral signalling elicitation (Brustolini *et al.*, 2015; Zorzatto *et al.*, 2015).

The current mechanistic model for the activation of NIK1‐mediated signalling holds that in response to virus infection, NIK1 undergoes homodimerization with itself or heterodimerization with an unknown receptor, promoting transphosphorylation at the essential threonine residue position 474 within the activation loop of the kinase (Fig. 1). Upon activation, NIK1‐mediated signalling mediates the phosphorylation of RIBOSOMAL PROTEIN L10 (RPL10), which in turn is redirected to the nucleus, where it interacts with L10‐INTERACTING MYB DOMAIN‐CONTAINING PROTEIN (LIMYB) to fully down‐regulate the expression of translational machinery‐related genes (Carvalho *et al.*, 2008c; Rocha *et al.*, 2008; Santos *et al.*, 2009; Zorzatto *et al.*, 2015). Therefore, the down‐regulation of the translation‐related genes is transcriptionally regulated through NIK1‐mediated formation of transcription‐repressing complexes by an association of phosphorylated RPL10 and LIMYB. The prolonged down‐regulation of the translational machinery leads to suppression of global translation. Plant DNA viruses cannot escape this translation regulatory mechanism of plant cells, and hence the viral mRNA is not efficiently translated, compromising the infection (Zorzatto *et al.*, 2015). Counteracting this activation mechanism, the begomovirus NSP binds to the kinase domain of NIK1 and prevents activation of the signalling pathway (Fontes *et al.*, 2004). Therefore, in compatible interactions, begomoviruses increase their pathogenicity to susceptible hosts by suppressing the activity of NIK1 kinase via interaction of the viral suppressor NSP, a reminiscent feature of PTI inactivation by pathogen suppressors.

Although the NIK1‐mediated antiviral responses are quite distinct from the PTI‐mediated antiviral defences, some similarities can be observed regarding the mechanism of activation and suppression of NIK1 and PRRs. First, NIK1 is presumably activated by ligand‐dependent complex formation that promotes phosphorylation and activation of the kinase domain. Phosphorylation of the functional analogues NIK1 Thr‐474, SERK1 Thr‐468 and BAK1 Thr‐455 is essential for receptor/coreceptor signalling, which may underscore a similar mechanism for activation (Santos *et al.*, 2009; Shah *et al.*, 2001; Wang *et al.*, 2005; Yun *et al.*, 2009). Second, this activation of NIK1 is induced by virus infection, which may provide virus‐derived PAMPs for NIK1‐mediated virus perception (Zorzatto *et al.*, 2015). Finally, NIK1 is suppressed by the viral effector NSP, which also functions as an Avr factor for ETI activation in resistant *Phaseolus vulgaris* genotypes (Garrido‐Ramirez *et al.*, 2000), linking a primary mechanism of antiviral immunity at the cell surface with ETI (Fig. 1). According to the zigzag evolutionary model of the two‐branch innate immune system, the activation of ETI in plant–virus interactions (NSP in resistant bean genotypes) is conceptually associated with PTI inhibition (NIK1 signalling) by a viral effector (NSP).

**Figure 2:**
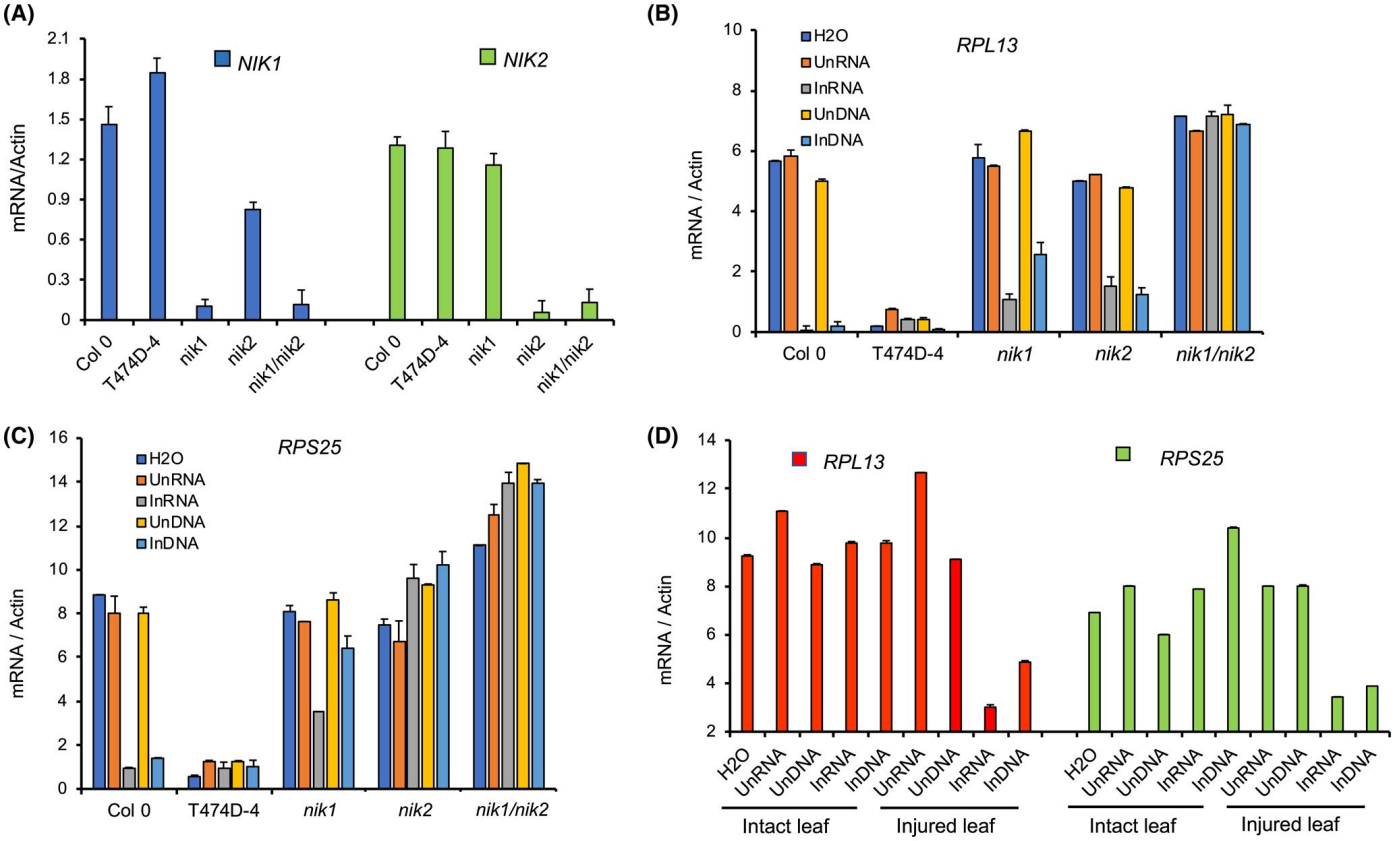
RNA and DNA prepared from begomovirus‐infected leaves activate the NIK1‐mediated antiviral signalling. (A) Single and double knockout lines of *NIK1* and *NIK2*. The expression of NIK1 or NIK2 in the leaves of Col‐0, *nik1‐1*, *nik2‐1* and *nik1‐1nk2‐1* lines was monitored by quantitative RT‐PCR. Mean ± 95% confidence intervals (*n* = 3) based on bootstrap resampling replicates of three independent experiments. (B) and (C) Begomovirus infection‐derived DNA and RNA down‐regulate RP genes, *RPL13* and *RPS25*, in a NIK1‐ and/or NIK2‐ependent manner. RP gene expression was monitored by qRT–PCR of RNA from Col‐0, *nik1‐1*, *nik2‐1* and *nik1‐1nk2‐1* lines treated with RNA or DNA from mock‐inoculated leaves (UnRNA and UnDNA) or CaLCuV‐infected leaves (InRNA and InDNA). Mean ± 95% confidence intervals (*n* = 3) based on bootstrap resampling replicates of three independent experiments. (D) Begomovirus infection‐derived RNA and DNA require mechanical injury in leaves to activate the NIK1/2‐mediated antiviral signalling. Intact, unwounded leaves and injured leaves were treated with RNA or DNA from CaLCuV‐infected leaves. After 3 h of treatment, *RP* gene expression was monitored by qRT–PCR.

A comparison of the transcriptome induced by ectopic expression of T474D and by begomovirus infection [*Cabbage leaf curl virus* (CaLCuV)/*Arabidopsis* and *Tomato yellow spot virus* (ToYCV)/Tomato] indicates that virus infection both activates and suppresses NIK1‐mediated antiviral signalling (Brustolini *et al.*, 2015; Zorzatto *et al.*, 2015). While the inhibition of the NIK1‐mediated antiviral signalling is due to the suppressive activity of the viral NSP, the mechanism by which the virus activates this antiviral response and the molecular bases for such elicitation are unknown. Here, we extended these studies by analysing whether begomovirus‐derived nucleic acids could act as PAMPs to activate NIK1. *Arabidopsis* wild‐type ecotype Columbia (Col‐0) plants at the seven‐leaf stage were inoculated by biolistic delivery with infectious clones of CaLCuV DNA‐A, and DNA‐B and the accumulation of viral DNA was monitored by PCR (Florentino *et al.*, 2006). Then, we used total RNA or total DNA isolated from infected plants subtracted from mock‐inoculated plants as sources for begomovirus‐derived nucleic acids. Leaf discs treated with RNA or DNA from virus‐infected plants were from the ecotype Columbia (Col‐0), the loss‐of‐function mutants *nik1‐1* (Fontes *et al.*, 2004), *nik2‐1* (Fontes *et al.*, 2004) and the double mutant *nik‐11nik2‐1* (Fig. 2A). *Arabidopsis* lines in which NIK1 was replaced by T474D (Zorzatto *et al.*, 2015) were also included as a positive control for NIK1 activation. The activation of the antiviral signal transduction was assayed by monitoring the down‐regulation of the ribosomal protein (RB) genes *RPL13* and *RPS25*, which have been shown to function as NIK1 activation‐associated marker genes (Brustolini *et al.*, 2015; Zorzatto *et al.*, 2015). Accordingly, ectopic expression of constitutively activated T474D in *nik1* null alleles caused the down‐regulation of both RP genes compared to the wild–type, Col‐0 line (Fig. 2B,C; Zorzatto *et al.*, 2015). In contrast, inactivation of both NIK1 and NIK2 in the double mutant *nik1‐1nik2‐1* released repression and caused up‐regulation of the RB genes, indicating that NIK1 and NIK2 are functionally paralogs for the NIK‐mediated repression of RB genes. These T474D‐ and *nik1nik2‐*mediated phenotypes recapitulate those resulting from overexpression and inactivation of the downstream transcriptional repressor LIMYB, respectively (Zorzatto *et al.*, 2015). Both DNA and RNA from infected plants (InDNA and InRNA) promoted down‐regulation of RP genes in Col‐0, as opposed to RNA and DNA from mock‐inoculated, uninfected plants (UnDNA and UnRNA, Fig. 2B,C). Inactivation of *NIK1* or *NIK2* caused a lower nucleic acid‐mediated repression of the RP genes compared with the level of repression in Col‐0. The down‐regulation of *RPL13* and *RPL25* was totally blocked in the double mutant *nik1nik2*. These results confirmed that NIK1 and NIK2 are functionally redundant and that the elicitation of NIK1‐mediated response by begomovirus‐derived DNA and RNA is dependent on NIK1 and/or NIK2. We also showed that virus‐derived RNA and DNA did not elicit NIK‐mediated antiviral signalling in unwounded leaves but instead were only effective if intact leaves were rubbed with abrasives before the treatment, which might have mechanically provided intracellular access for viral PAMPs (Fig. 2D).

Our findings suggest that begomovirus‐derived RNA and DNA function as viral PAMPs, which elicit the NIK‐mediated antiviral signalling in a NIK1‐ and/or NIK2‐dependent manner. However, the nature and origin of the viral PAMPs, how viruses are sensed by NIKs extracellularly, and whether NIK1 and NIK2 function as coreceptors or genuine PRRs remain to be determined.

### Mechanistic hypotheses for the perception of viral PAMPs by RLKs at the cell surface

Because viruses are obligate intracellular parasites, viral PAMPs are expected to be produced intracellularly and there is no evidence that virus‐derived nucleic acids can reach the apoplast to be sensed directly by the LRR extracellular domain of NIKs and SERKs or associated PRRs. It is possible that plasma membrane anchored receptors sense viral PAMPs through their intracellular domain, thus antiviral PTI would be activated by viral PAMP‐induced receptor–coreceptor dimerization via the kinase domain. The intracellular mammalian protein kinase RNA‐activated (PKR) undergoes dsRNA ligand‐mediated dimerization and activation upon direct association of viral dsRNA produced during infection with its kinase domain (Balachandran *et al.*, 2000).

Alternatively, the microsomal recycling pathway may provide the opportunistic colocalization for NIKs, SERKs and PRRs with viral PAMPs. The movement proteins (MPs) of plant viruses use the endocytic recycling pathway to move viral RNA– and viral DNA–protein complexes to the plasmodesmata for the MP‐assisted cell‐to‐cell spread of the virus (Lewis and Lazarowitz, 2010). Plant PRRs have also been shown to be internalized via endosomes (Mbengue *et al.*, 2016). Therefore, the specific perception of a virus by PRRs would rely on the opportunistic subcellular colocalization of internalized PRRs and the MP‐associated viral genome in host cells. Consistent with both alternatives, virus‐derived nucleic acids did not activate the NIK1‐mediated antiviral signalling in intact, unwounded leaves but instead required the leaves to be mechanically injured, which may have provided the intracellular access for viral PAMPs. Complementary experiments by expressing truncated, extracellular LRR‐less NIKs and/or SERKs, which remain associated with the plasma membrane, may facilitate addressing this issue.

Although this endocytic recycling pathway is an attractive hypothesis for virus perception by plant PRRs, similar to mammalian endosomal TLRs, it may not be possible to synchronize PTI activation as a primary, early response to pathogen attack, with the later event of virus movement to adjacent cells. Therefore, only with the identification of the origin and nature of virus‐derived PAMPs and the characterization of nucleic acid‐sensing PRRs will it be possible to address these possibilities for virus perception by plasma membrane‐anchored RLKs.

## Conclusion

Recent studies with SERK‐like coreceptors and known PRRs, which function in plant innate immunity, have demonstrated that the classical PTI is part of the antiviral defence arsenal of plant cells. Several components of classic PTI, including the co‐receptor SERKs, BAK1 and SERK1, and the MAPK4 negative regulator, in addition to common immune responses, co‐exist as part of antiviral PTI. As for viral PAMPs, dsRNAs isolated from virus‐infected plants have been demonstrated to induce typical PTI responses dependent on the coreceptor SERK1. Likewise, begomovirus‐derived nucleic acids were shown here to induce the NIK1‐mediated antiviral signalling in a NIK1‐ and NIK2‐dependent manner and were included as a piece of new complementary evidence for this review. However, the nature and origin of the nucleic acid PAMPs have not been deciphered, and the nucleic acid sensors have yet to be identified. Furthermore, as viruses are intracellular obligate parasites and may not have access to the apoplast, it remains to be determined how PRRs would sense viruses and viral PAMPS extracellularly. The identification of the origin and nature of virus‐derived nucleic acid PAMPs and the discovery of the cognate PRRs will shed light on the mechanism of antiviral PTI activation and its role in resistance against viruses.

## Conflict of Interest

The authors declare no conflict of interest.

## References

[mpp12816-bib-0001] Balachandran, S. , Roberts, P.C. , Brown, L.E. , Truong, H. , Pattnaik, A.K. , Archer, D.R. and Barber, G.N. (2000) Essential role for the dsRNA‐dependent protein kinase PKR in innate immunity to viral infection. Immunity, 13, 129–141. 10933401 10.1016/s1074-7613(00)00014-5

[mpp12816-bib-0002] Botos, I. , Segal, D.M. and Davies, D.R. (2011) The structural biology of Toll‐like receptors. Structure, 19, 447–459. 21481769 10.1016/j.str.2011.02.004 PMC3075535

[mpp12816-bib-0003] Brustolini, O.J.B. , Machado, J.P.B. , Condori‐Apfata, J.A. , Coco, D. , Deguchi, M. , Loriato, V.A. , Pereira, W.A. , Alfenas‐Zerbini, P. , Zerbini, F.M. , Inoue‐Nagata, A.K. , Santos, A.A. , Chory, J. , Silva, F.F. and Fontes, E.P.B. (2015) Sustained NIK‐mediated antiviral signalling confers broad‐spectrum tolerance to begomoviruses in cultivated plants. Plant Biotechnol. J. 13, 1300–1311. 25688422 10.1111/pbi.12349 PMC4857726

[mpp12816-bib-0004] Calil, I.P. and Fontes, E.P.B. (2017) Plant immunity against viruses: antiviral immune receptors in focus. Ann. Bot. 118, 200–213. 10.1093/aob/mcw200 PMC5604577 27780814

[mpp12816-bib-0005] Carvalho, C.M. , Fontenelle, M.R. , Florentino, L.H. , Santos, A.A. , Zerbini, F.M. and Fontes, E.P.B. (2008a) A novel nucleocytoplasmic traffic GTPase identified as a functional target of the bipartite geminivirus nuclear shuttle protein. Plant J. 5, 869–880. 10.1111/j.1365-313X.2008.03556.x 18489709

[mpp12816-bib-0006] Carvalho, C.M. , Machado, J.P. , Zerbini, F.M. and Fontes, E.P. (2008b) NSP‐interacting GTPase: a cytosolic protein as cofactor for nuclear shuttle proteins. Plant Signal. Behav. 3, 752–754. 19704847 10.4161/psb.3.9.6641 PMC2634578

[mpp12816-bib-0007] Carvalho, C.M. , Santos, A.A. , Pires, S.R. , Rocha, C.S. , Saraiva, D.I. , Machado, J.P.B. , Mattos, E.C. , Fietto, L.G. and Fontes, E.P.B. (2008c) Regulated nuclear trafficking of rpL10A mediated by NIK1 represents a defense strategy of plant cells against virus. PLoS Pathog. 4, e1000247. 19112492 10.1371/journal.ppat.1000247 PMC2597721

[mpp12816-bib-0008] Choi, H.W. and Klessig, D.F. (2016) DAMPs, MAMPs, and NAMPs in plant innate immunity. BMC Plant Biol. 16, 232. 27782807 10.1186/s12870-016-0921-2 PMC5080799

[mpp12816-bib-0009] Decroocq, V. , Salvador, B. , Sicard, O. , Glasa, M. , Cosson, P. , Svanella‐Dumas, L. , Revers, F. , García, J.A. and Candresse, T. (2009) The determinant of potyvirus ability to overcome the RTM resistance of *Arabidopsis thaliana* maps to the N‐terminal region of the coat protein. Mol. Plant Microbe Interact. 22, 1302–1311. 19737103 10.1094/MPMI-22-10-1302

[mpp12816-bib-0010] Florentino, L.H. , Santos, A.A. , Fontenelle, M.R. , Pinheiro, G.L. , Zerbini, F.M. , Baracat‐Pereira, M.C. and Fontes, E.P.B. (2006) A PERK‐like receptor kinase interacts with the geminivirus nuclear shuttle protein and potentiates viral infection. J. Virol. 80, 6648–6656. 16775352 10.1128/JVI.00173-06 PMC1488943

[mpp12816-bib-0011] Fontes, E.P.B. , Santos, A.A. , Luz, D.F. , Waclawovsky, A.J. and Chory, J. (2004) The geminivirus nuclear shuttle protein is a virulence factor that suppresses transmembrane receptor kinase activity. Genes Dev. 18, 2545–2556. 15489295 10.1101/gad.1245904 PMC529541

[mpp12816-bib-0012] Garrido‐Ramirez, E.R. , Sudarshana, M.R. , Lucas, W.J. and Gilbertson, R.L. (2000) Bean dwarf mosaic virus BV1 protein is a determinant of the hypersensitive response and avirulence in phaseolus vulgaris. Mol. Plant Microbe Interact. 13, 1184–1194. 11059485 10.1094/MPMI.2000.13.11.1184

[mpp12816-bib-0013] Gouveia, B.C. , Calil, I.P. , Machado, J.P.B. , Santos, A.A. and Fontes, E.P.B. (2017) Immune receptors and co‐receptors in antiviral innate immunity in plants. Front. Microbiol. 7, 2139. 28105028 10.3389/fmicb.2016.02139 PMC5214455

[mpp12816-bib-0014] Iriti, M. and Varoni, E.M. (2015) Chitosan‐induced antiviral activity and innate immunity in plants. Environ. Sci. Pollut. Res. Int. 22, 2935–2944. 25226839 10.1007/s11356-014-3571-7

[mpp12816-bib-0015] Jensen, S. and Thomsen, A.R. (2012) Sensing of RNA viruses: a review of innate immune receptors involved in recognizing RNA virus invasion. J. Virol. 86, 2900–2910. 22258243 10.1128/JVI.05738-11 PMC3302314

[mpp12816-bib-0016] Jones, J.D. and Dangl, J.L. (2006) The plant immune system. Nature, 444, 323–329. 17108957 10.1038/nature05286

[mpp12816-bib-0017] Kong, J. , Wei, M. , Li, G. , Lei, R. , Qiu, Y. , Wang, C. , Li, Z.‐H. and Zhu, S. (2018) The cucumber mosaic virus movement protein suppresses PAMP‐triggered immune responses in Arabidopsis and tobacco. Biochem. Biophys. Res. Comm. 498, 395–401. 29407169 10.1016/j.bbrc.2018.01.072

[mpp12816-bib-0018] Korner, C.J. , Klauser, D. , Niehl, A. , Dominguez‐Ferreras, A. , Chinchilla, D. , Boller, T. , Heinlein, M. and Hann, D.R. (2013) The immunity regulator BAK1 contributes to resistance against diverse RNA viruses. Mol. Plant Microbe Interact. 26, 1271–1280. 23902263 10.1094/MPMI-06-13-0179-R

[mpp12816-bib-0019] Lewis, J.D. and Lazarowitz, S.G. (2010) *Arabidopsis* synaptotagmin SYTA regulates endocytosis and virus movement protein cell‐to‐cell transport. Proc. Natl. Acad. Sci. USA, 107, 2491–2496. 20133785 10.1073/pnas.0909080107 PMC2823903

[mpp12816-bib-0020] Liu, J.Z. , Horstman, H.D. , Braun, E. , Graham, M.A. , Zhang, C. , Navarre, D. , Qiu, W.L. , Lee, Y. , Nettleton, D. , Hill, J.H. and Whitham, S.A. (2011) Soybean homologs of MPK4 negatively regulate defense responses and positively regulate growth and development. Plant Physiol. 157, 1363–1378. 21878550 10.1104/pp.111.185686 PMC3252160

[mpp12816-bib-0021] Ma, X. , Xu, G. , He, P. and Shan, L. (2016) SERKing coreceptors for Receptors. Trends Plant Sci. 21, 1017–1033. 27660030 10.1016/j.tplants.2016.08.014

[mpp12816-bib-0022] Machado, J.P.B. , Brustolini, O.J.B. , Mendes, G.C. , Santos, A.A. and Fontes, E.P.B. (2015) NIK1, a host factor specialized in antiviral defense or a novel general regulator of plant immunity? BioEssays, 37, 1236–1242. 26335701 10.1002/bies.201500066

[mpp12816-bib-0023] Machado, J.P.B. , Calil, I.P. , Santos, A.A. and Fontes, E.P.B. (2017) Translational control in plant antiviral immunity. Genet. Mol. Biol. 40, 292–304. 28199446 10.1590/1678-4685-GMB-2016-0092 PMC5452134

[mpp12816-bib-0024] Mariano, A.C. , Andrade, M.O. , Santos, A.A. , Carolino, S.M.B. , Oliveira, M.L. , Baracat‐Pereira, M.C. , Brommonshenkel, S.H. and Fontes, E.P.B. (2004) Identification of a novel receptor‐like protein kinase that interacts with a geminivirus nuclear shuttle protein. Virology, 318, 24–31. 14972531 10.1016/j.virol.2003.09.038

[mpp12816-bib-0025] Mbengue, M. , Bourdais, G. , Gervasi, F. , Beck, M. , Zhou, J. , Spallek, T. , Bartels, S. , Boller, T. , Ueda, T. , Kuhn, H. and Robatzek, S. (2016) Clathrin‐dependent endocytosis is required for immunity mediated by pattern recognition receptor kinases. Proc. Natl. Acad. Sci. USA, 113, 11034–11039. 27651493 10.1073/pnas.1606004113 PMC5047200

[mpp12816-bib-0026] Moon, J.Y. and Park, J.M. (2016) Cross‐talk in viral defense signaling in plants. Front. Microbiol. 7, 2068. 28066385 10.3389/fmicb.2016.02068 PMC5174109

[mpp12816-bib-0027] Nicaise, V. and Candresse, T. (2017) Plum pox virus capsid protein suppresses plant pathogen‐associated molecular pattern (PAMP)‐triggered immunity. Mol. Plant Pathol. 18, 878–886. 27301551 10.1111/mpp.12447 PMC6638313

[mpp12816-bib-0028] Niehl, A. , Wyrsch, I. , Boller, T. and Heinlein, M. (2016) Double‐stranded RNAs induce a pattern‐triggered immune signaling pathway in plants. New Phytol. 211, 1008–1019. 27030513 10.1111/nph.13944

[mpp12816-bib-0029] Rocha, C.S. , Santos, A.A. , Machado, J.P.B. and Fontes, E.P.B. (2008) The ribosomal protein L10/QM‐like protein is a component of the NIK‐mediated antiviral signaling. Virology, 380, 165–169. 18789471 10.1016/j.virol.2008.08.005

[mpp12816-bib-0030] Sakamoto, T. , Deguchi, M. , Brustolini, O.J.B. , Santos, A.A. , Silva, F.F. and Fontes, E.P.B. (2012) The tomato RLK superfamily: phylogeny and functional predictions about the role of the LRRII‐RLK subfamily in antiviral defense. BMC Plant Biol. 12, 229. 23198823 10.1186/1471-2229-12-229 PMC3552996

[mpp12816-bib-0031] Santos, A.A. , Carvalho, C.M. , Florentino, L.H. , Ramos, H.J.O. and Fontes, E.P. (2009) Conserved threonine residues within the A‐loop of the receptor NIK differentially regulate the kinase function required for antiviral signaling. PLoS ONE, 4, e5781. 19492062 10.1371/journal.pone.0005781 PMC2686266

[mpp12816-bib-0032] Santos, A.A. , Lopes, K.V.G. , Apfata, J.A.C. and Fontes, E.P.B. (2010) NSP‐interacting kinase, NIK: a transducer of plant defence signalling. J. Exp. Bot. 61, 3839–3845. 20624762 10.1093/jxb/erq219

[mpp12816-bib-0033] Shah, K. , Vervoort, J. and de Vries, S.C. (2001) Role of threonines in the *Arabidopsis thaliana* somatic embryogenesis receptor kinase 1 activation loop in phosphorylation. J. Biol. Chem. 276, 41263–41269. 11509554 10.1074/jbc.M102381200

[mpp12816-bib-0034] Silva, J.C.F. , Carvalho, T.F.M. , Basso, M.F. , Deguchi, M. , Pereira, W.A. , Sobrinho, R.R. , Vidigal, P.M.P. , Brustolini, O.J.B. , Silva, F.F. , Dal‐Bianco, M. , Fontes, R.L.F. , Santos, A.A. , Zerbini, F.M. , Cerqueira, F.R. and Fontes, E.P.B. (2017) Geminivirus data warehouse: a database enriched with machine learning approaches. BMC Bioinformatics, 18, 240. 28476106 10.1186/s12859-017-1646-4 PMC5420152

[mpp12816-bib-0035] Wang, Y. and Wang, Y. (2018) Trick or treat: microbial pathogens evolved apoplastic effectors modulating plant susceptibility to infection. Mol. Plant‐Microbe Interact. 3, 6–12. 10.1094/MPMI-07-17-0177-FI 29090656

[mpp12816-bib-0036] Wang, X. , Goshe, M.B. , Soderblom, E.J. , Phinney, B.S. , Kuchar, J.A. , Li, J. , Asami, T. , Yoshida, S. , Huber, S.C. and Clouse, S.D. (2005) Identification and functional analysis of in vivo phosphorylation sites of the Arabidopsis BRASSINOSTEROID‐INSENSITIVE1 receptor kinase. Plant Cell, 17, 1685–1703. 15894717 10.1105/tpc.105.031393 PMC1143070

[mpp12816-bib-0037] Yang, H. , Gou, X. , He, K. , Xi, D. , Du, J. , Lin, H. and Li, J. (2010) BAK1 and BKK1 in *Arabidopsis thaliana* confer reduced susceptibility to *Turnip crinkle virus* . Eur. J. Plant Pathol. 127, 149–156.

[mpp12816-bib-0038] Yokota, S.I. , Okabayashi, T. and Fujii, N. (2010) The battle between virus and host: modulation of Toll‐like receptor signaling pathways by virus infection. Mediators Inflamm. 2010, 184323. 10.1155/2010/184328 PMC2903949 20672047

[mpp12816-bib-0039] Yun, H.S. , Bae, Y.H. , Lee, Y.J. , Chang, S.C. , Kim, S.K. , Li, J. and Nam, K.H. (2009) Analysis of phosphorylation of the BAK1/BRI1 complex in Arabidopsis reveals amino acid residues critical for receptor formation and activation of BR signaling. Mol. Cells, 27, 183–190. 19277500 10.1007/s10059-009-0023-1

[mpp12816-bib-0040] Zhang, X.S. , Choi, J.H. , Heinz, J. and Chetty, C.S. (2006) Domain‐specific positive selection contributes to the evolution of Arabidopsis leucine‐rich repeat receptor‐like kinase (LRR RLK) genes. J. Mol. Evol. 63, 612–621. 17031460 10.1007/s00239-005-0187-z

[mpp12816-bib-0041] Zorzatto, C. , Machado, J.P.B. , Lopes, K.V.G. , Nascimento, K.J.T. , Pereira, W.A. , Brustolini, O.J.B. , Reis, P.A.B. , Calil, I.P. , Deguchi, M. , Sachetto‐Martins, G. , Gouveia, B.C. , Loriato, V.A.P. , Silva, M.A.C. , Silva, F.F. , Santos, A.A. , Chory, J. and Fontes, E.P.B. (2015) NIK1‐mediated translation suppression functions as a plant antiviral immunity mechanism. Nature, 520, 679–682. 25707794 10.1038/nature14171 PMC4779052

[mpp12816-bib-0042] Zvereva, A.S. , Golyaev, V. , Turco, S. , Gubaeva, E.G. , Rajeswaran, R. , Schepetilnikov, M.V. , Srour, O. , Ryabova, L.A. , Bolle, T. and Pooggin, M.M. (2016) Viral protein suppresses oxidative burst and salicylic acid‐dependent autophagy and facilitates bacterial growth on virus‐infected plants. New Phytol. 211, 1020e1034. 27120694 10.1111/nph.13967

